# Whole Exome Sequencing of Hemiplegic Migraine Patients Shows an Increased Burden of Missense Variants in *CACNA1H* and *CACNA1I* Genes

**DOI:** 10.1007/s12035-023-03255-5

**Published:** 2023-02-14

**Authors:** Neven Maksemous, Aster V. E. Harder, Omar Ibrahim, Lisanne S. Vijfhuizen, Heidi Sutherland, Nadine Pelzer, Irene de Boer, Gisela M. Terwindt, Rodney A. Lea, Arn M. J. M. van den Maagdenberg, Lyn R. Griffiths

**Affiliations:** 1 Genomics Research Centre, Centre for Genomics and Personalised Health, School of Biomedical Sciences, Queensland University of Technology, 60 Musk Ave, Brisbane, QLD 4059 Australia; 2 Department of Human Genetics, Leiden University Medical Centre, Leiden, the Netherlands; 3 Department of Neurology, Leiden University Medical Centre, Leiden, the Netherlands

**Keywords:** Hemiplegic migraine, *CACNA1x*, Headache, Burden-testing

## Abstract

Hemiplegic migraine (HM) is a rare subtype of migraine with aura. Given that causal missense mutations in the voltage-gated calcium channel α1A subunit gene *CACNA1A* have been identified in a subset of HM patients, we investigated whether HM patients without a mutation have an increased burden of such variants in the “*CACNA1x* gene family”. Whole exome sequencing data of an Australian cohort of unrelated HM patients (*n* = 184), along with public data from gnomAD, as controls, was used to assess the burden of missense variants in *CACNA1x* genes. We performed both a variant and a subject burden test. We found a significant burden for the number of variants in *CACNA1E* (*p* = 1.3 × 10^−4^), *CACNA1H* (*p* < 2.2 × 10^−16^) and *CACNA1I* (*p* < 2.2 × 10^−16^). There was also a significant burden of subjects with missense variants in *CACNA1E* (*p* = 6.2 × 10^−3^), *CACNA1H* (*p* < 2.2 × 10^−16^) and *CACNA1I* (*p* < 2.2 × 10^−16^). Both the number of variants and number of subjects were replicated for *CACNA1H* (*p* = 3.5 × 10^−8^; *p* = 0.012) and *CACNA1I* (*p* = 0.019, *p* = 0.044), respectively, in a Dutch clinical HM cohort (*n* = 32), albeit that *CACNA1I* did not remain significant after multiple testing correction. Our data suggest that HM, in the absence of a single causal mutation, is a complex trait, in which an increased burden of missense variants in *CACNA1H* and *CACNA1I* may contribute to the risk of disease.

**Supplementary Information:**

The online version contains supplementary material available at 10.1007/s12035-023-03255-5.

## Background

Hemiplegic migraine (HM) is a rare subtype of migraine with aura with attacks that are associated with motor weakness or hemiplegia during the aura phase [1]. HM is clinically and genetically heterogeneous [2–4] and can be subdivided into familial hemiplegic migraine (FHM) and sporadic hemiplegic migraine (SHM), distinguished by having a positive or negative family history for HM, respectively [1].

A subset of HM patients exhibits an autosomal dominant phenotype with single high-penetrant causal mutations present in ion transport genes *CACNA1A*, *ATP1A2* or *SCN1A* [5–7]. However, in many HM patients, no such pathogenic mutation has been detected [8, 9]. Whereas evidence is accumulating that loss-of-function mutations in *PRRT2* [10], a key component of the Ca^2+^-dependent neurotransmitter release machinery [11], are involved in HM, the gene more likely acts as a modifier of disease [12]. This suggests that HM, in a set of patients, may be regarded a complex disorder with multiple genetic factors contributing to the phenotype. Most relevant, a Finnish polygenic risk score study of genome-wide association study (GWAS) data has shown that HM patients without a high-penetrant disease-causing mutation in a known HM gene carry an excess of common (frequency > 1%) variants compared to patients suffering from common (complex) migraine subtypes [13].

Following along this line of evidence, it has been hypothesised that complex disorders can be the result of an accumulation of genetic variants in a disease pathway, where the crossing of a certain threshold leads to disease [14]. Moreover, current evidence indicates that complex traits are likely to be underpinned by a combination of multiple common and rare variants [15–17]. Here we set out to investigate the contribution of modulatory genetic effects that can be studied through testing the synergistic burden of (functional) variants, best annotated as missense variants, rather than a single causative mutation. Burden can be regarded as an accumulation of variants that are more often present in cases compared to controls. We hypothesise here that the burden of missense variants in certain ion channel genes might be involved in the disease pathology of HM.

**Fig. 1:**
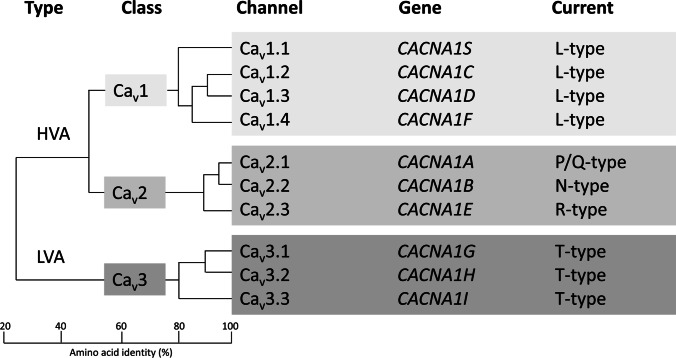
The voltage-gated calcium channel (VGCC) family of proteins. The α1 subunits can be divided into three subclasses according to their amino acid sequence identity, as shown in the dendrogram. Ca_V_1 and Ca_V_2 channels are high-voltage-activated (HVA), whereas Ca_V_3 channels are low-voltage-activated (LVA). The genes encoding the respective α1 subunit are provided as well as the type of current the respective channel type produces. The schematic is based upon Perez-Reyes and Dolphin [22, 23]

*CACNA1A* was the first HM gene discovered and encodes the pore‐forming α_1A_ subunit of the neuronal voltage‐gated calcium channel (VGCC) Ca_V_2.1 (P/Q‐type) [5, 18]. Ca_V_2.1 channels are predominantly localised at presynaptic terminals and play a prominent role in controlling neurotransmitter release at most synapses of the nervous system [19–21]. *CACNA1A* is a member of a family of rather conserved α1 subunit genes, hereafter referred to as “*CACNA1x*”, which are part of VGCCs that are classified as either high-voltage-activated (HVA) or low-voltage-activated (LVA) channels that are present on the membranes of excitable cells (Fig. 1) [22, 23]. Ca_V_ channels are typically composed of multiple subunits, namely an α_1_, a β, an α2/δ and a γ subunit. An α_1_ subunit has 24 transmembrane segments and forms the pore through which calcium ions pass into the cell. The main characteristic of the various Ca_V_ channel types is primarily determined by the type of α1 subunit, so the presence of either α_1A_, α_1B_, α_1C_, α_1D_, α_1E_, α_1F_, α_1G_, α_1H_, α_1I_ or α_1S_. Given the important functions of Ca_V_ channels, it is not surprising that genetic variation in *CACNA1x* genes is not well-tolerated; the residual variation intolerance scores for these genes are high (Table 1) [24].

**Table 1:** Intolerance scores *CACNA1x* genes

Gene	RVIS	%ExAC RVIS	%ExAC Lof FDR	%ExAC v2 RVIS	Edge case (%OE-ratio)
*CACNA1A*	− 1.78 (2.27%)	1.68%	1.35 × 10^−10^	− 2.20 (2.45%)	*N* (4.41%)
*CACNA1B*	NA (NA)	0.79%	1.48 × 10^−5^	− 2.34 (2.17%)	*N* (10.98%)
*CACNA1C*	− 2.09 (1.57%)	3.51%	9.23 × 10^−8^	− 1.53 (5.77%)	*N* (4.20%)
*CACNA1D*	− 3.51 (0.32%)	0.39%	1.64 × 10^−13^	− 3.37 (0.72%)	*N* (11.785)
*CACNA1E*	− 2.71 (0.71%)	3.34%	8.29 × 10^−18^	− 1.88 (3.59%)	*N* (5.73%)
*CACNA1F*	− 0.83 (11.53%)	12.28%	1.03 × 10^−4^	0.02 (52.82%)	*N* (45.93%)
*CACNA1G*	− 2.37 (1.11%)	1%	2.45 × 10^−8^	− 2.08 (2.85%)	*N* (13.56%)
*CACNA1H*	− 2.06 (1.63%)	25.99%	1.79 × 10^−9^	− 0.03 (48.18%)	*N* (13.68%)
*CACNA1I*	− 0.83 (11.55%)	3.96%	4.29 × 10^−6^	− 1.90 (3.52%)	*N* (7.79%)
*CACNA1S*	− 1.21 (5.71%)	61.36%	8.21 × 10^−5^	0.52 (72.65%)	*N* (41.68%)

*RVIS*, residual variation intolerance score (is a gene score based module intended to help in the interpretation of human sequence data); %ExAC RVIS, RVIS v3, constructed on the ExAC data release; ExAC LoF FDR, FDR-adjusted *p*-value reflects the significance of the departure from the expected rate of LoF variants; %ExAC v2 RVIS, RVIS v4, constructed on the ExAC v2 data release; *Edge Case*, edge case genes. The OE ratio is based on ExAC v2 data release

The expression of *CACNA1x* genes varies considerably and, with the exception of *CACNA1S*, all are expressed in the brain [25]. In addition to *CACNA1A* being a well-known HM gene, there have been rare reports on other *CACNA1x* genes possibly involved in HM-relevant phenotypes. For instance, a link between hemiplegic migraine and brain stem aura migraine has been suggested for *CACNA1E* [26], and headache with neurological deficits and cerebrospinal fluid lymphocytosis (HaNDL), a headache syndrome with much phenotypic resemblance to HM, has been linked to the occurrence of antibodies against *CACNA1H* [27], a gene implicated in childhood epilepsy although this has recently been debated [28]. Furthermore, using a systems genetics approach, Rasmussen et al. [29] identified *CACNA1B* as one of the genes commonly mutated in migraine families. Finally, *CACNA1A* was identified as a risk locus for common migraine, as well as being one of the three genes specific for migraine with aura [30]. Although no definite proof for a causal link was provided in any of these cases, the existing data can be regarded as supportive evidence for a spectrum ranging from rare to common variants contributing to certain extent to the risk for both common and hemiplegic migraine. This variety of observed variants makes the family of *CACNA1x* genes an interesting candidate for burden testing in HM, with relevance, foremost, to patients with a complex genetic basis.

Whole exome sequencing (WES) enables comprehensive exploration of missense variants and investigation of their role in complex traits. When considering that these missense variants are unlikely to be causing HM as monogenic factors as occurs for patients with specific *CACNA1A*, *ATP1A2* and *SCN1A* mutations [5–7], burden testing is a potential way to explore their potential synergistic effect on increasing HM disease risk. Burden testing typically requires a set of qualifying variants, often rare, protein-altering variants in the case of a monogenic condition. However, following on from the hypothesis that HM seems not a monogenic disorder in *all* patients, the accumulation of both rare and common protein-altering variants may be relevant in terms of disease susceptibility. The use of large publicly available WES datasets from general population controls can be incorporated in burden testing to gain more reliable estimates of gene-wide susceptibility.

We hypothesise that the burden of multiple missense variants in *CACNA1x* genes increases the risk for HM, burden being the aggregation of both rare and common variants as well as the increased presence of a variant in cases compared to controls. To this end, we here used WES data from a large Australian HM patient cohort to identify missense variants in eight calcium channel genes (*CACNA1A*, *-B*, *-C*, *-D*, *-E*, *-G*, *-H* and *-I*) and determined whether the aggregated effect of the variants across the genes was higher than observed in general population controls. Results were validated in an independent Dutch clinical HM cohort.

## Methods

### Study Cohorts

The study consisted of two cohorts of HM patients: an Australian cohort of 184 patients (discovery cohort) and a Dutch cohort of 32 patients (replication cohort). Importantly, patients were a priori excluded in case a pathogenic mutation was present in one of the three HM genes (*CACNA1A*, *ATP1A2* and *SCN1A*) or HM-related genes with mutations confirmed by Sanger sequencing [9, 31].

#### Australian Cohort

The Australian cohort was selected out of over 300 patients that had been referred to the Genomics Research Centre (GRC) Diagnostic Clinic for genetic diagnostic testing after a suspected diagnosis of HM from the referring neurologist. From this cohort, a subset of 184 (122 females and 62 males) unrelated individuals tested negative for known HM gene mutations (*CACNA1A*, *ATP1A2* and *SCN1A*) and HM-related genes [9, 31]. All cases consented to genetic testing with their doctors, as required under current regulations. Positive family history was reported for 25% of the cases; 5% were reported as SHM, while family information was not available for the remainder of cases.

DNA was extracted from blood samples using QIAGEN QIAamp DNA Mini Kit as per the manufacturers’ instructions. Next generation sequencing (NGS) libraries for WES were constructed using the Ion AmpliSeq™ Exome RDY library kits (ThermoFisher Scientific, Waltham, MA, USA) according to the manufacturer’s protocol. The Ion Chef was used to load sample libraries (barcoded fragments of 200 bp). WES was performed in the Genomics Research Centre (GRC), Australia via the Ion Proton and GeneStudio S5 plus (ThermoFisher Scientific) instruments using default settings for Ion AmpliSeq Exome RDY Kit 4 × 2 (Thermo Fisher Scientific). The study was conducted in accordance with the Declaration of Helsinki, and the protocol was approved by the Human Research Ethics Committee of the Queensland University of Technology (approval number: 1800000611).

#### Dutch Cohort

The cohort consisted of 32 patients (22 females and 10 males) with FHM/SHM according to ICHD-3 criteria [32]. Patients were selected from the Leiden Headache Centre at the Leiden University Medical Centre (LUMC), and contained patients (i) seen in person by experienced headache clinicians or research physicians or (ii) referred from elsewhere for clinical genetic research with records being evaluated and clinical diagnosis confirmed by GMT, NP and IdB [4]. All patients were from different families and did not have a known pathogenic mutation in one of the three HM genes. The study was approved by the Medical Ethics Committee of LUMC and all participants provided informed consent.

Genomic DNA was extracted from peripheral blood leukocytes according to the standard salting-out protocol [33]. WES was performed using in-house sequencing facility (Leiden Genome Technology Centre; URL: lgtc.nl) or outsourced to the Beijing Genomics Institute sequencing facility (URL: bgi.com). In brief, for the LGCT, coding sequences in the DNA were enriched using the SureSelect Human All Exon 50 Mb kit (Agilent Technologies, Santa Clara, CA, USA). Following sequence capture and amplification, fragments were sequenced using the Illumina HiSeq2000 platform (San Diego, CA, USA).

#### Controls

As a control dataset, we used summary statistics from gnomAD (see below in the paragraph on TRAPD methods). The gnomAD database was chosen as it consists of a large number of individuals and contains a detailed catalogue of exome-wide genetic variation. Furthermore, gnomAD provided ancestry information. The gnomAD database contains exome variant summary statistics for 56,885 non-Finnish Europeans, with a female-to-male ratio of ~ 1.27:1, depending on the available genotypes at each specific locus. As HM is a very rare disorder with a prevalence of 0.01% [34], confounding effects due to the presence of HM patients in the control group were deemed to be negligible.

### Whole Exome Sequencing and Quality Control

#### Australian Cohort

Following WES, the Ion Torrent Server was used to generate quality metrics, align reads to the Human Genome 19 (Hg19), and the Ion Torrent Variant Caller (TVC) was used to call sequence variants and produce variant calling format (VCF) files.

#### Dutch Cohort

Following sequencing, the sequence reads were aligned to the UCSC Genome Browser hg19 reference sequence using the Burrows-Wheeler Alignment tool [35]. The generated BAM files were subsequently converted to VCF files using BCFtools [36].

### Single-Variant Analysis

Prior to performing burden testing, all variants were assessed to determine whether there were obvious, high-penetrant disease-causing mutations detected outside of the known HM genes that could cause HM in patients of either cohort. In the absence of such pathogenic mutation, individual missense variants in all the *CACNA1x* genes were assessed for patients of the Australian cohort. For the Dutch cohort, only those missense variants present in TRAPD-associated *CACNA1x* genes were investigated.

### Burden Testing

#### Variants Pre-processing All Cohorts

As VCFs were exported from different platforms, the respective analyses had to be unified. The first step for both cohorts was to normalise VCFs using BCFtool; this ensures that any platform-specific formatting differences are removed and also expands multi-allelic variants [36]. VCFs were merged for each cohort using vcftools, and variants with average read depth coverage below × 10 were excluded using either BCFtools or the snpEff program [36, 37]. For both cohorts, the coding exons of the *CACNA1x* genes were included with a 5-bp pad on either side of the exon. New VCFs (one merged for each cohort) were annotated with VEP Ensembl [38]. For the Dutch cohort as an extra quality control step, only those variants with a quality-by-depth (QD) score > 4 were taken forward.

#### Selection of Qualifying Variants

To determine the number of variants, we selected “qualifying variants” being variants that meet the criteria of inclusion. Only those variants classified (annotated) as missense variants were considered as “qualifying variants”. The number of individuals in the case cohort who carried at least one “qualifying variant” in that gene and the total number of variants were used in the analysis. For the gnomAD control dataset, only summary statistics were available. Therefore, to approximate the number of control subjects carrying at least one qualifying variant in a given gene, the allele counts for all qualifying variants in that gene were summed. This summation-based approximation probably is an overestimation as it is likely that some individuals carry multiple variants in the same gene. Contrary to rare variant analysis where only the locations of the qualifying variant in cases are used for controls, we selected all variants across the entire gene in controls, in the same way as what was done for the cases. As a result, we had a total number of all missense variants per *CACNA1x* gene in both cases and controls. These “qualifying variants” for both the case and the control cohort were compared. Insertions and deletions (Indels) were not included in the analysis due to their higher percentage of sequencing artefacts, especially given the differing sequencing platforms used across cohorts.

#### Multiple-Variant Burden Testing of *CACNA1x* Genes

Gene-based burden testing was performed for all variants that met the quality filters, which are referred to as “qualifying variants”, using adaptation on the TRAPD test (Testing Rare vAriants using Public Data) [39]. TRAPD was chosen because the control dataset consisted of summary data rather than individual-level genotype data as well as for its approach to collate variants which mitigates the statistical drawbacks of burden testing per variant or per individual. The TRAPD test was implemented to determine whether *CACNA1x* genes and subjects carried a significant burden of missense variants in cases. TRAPD produces counts of “collapsed” variant groups across each gene and for the respective case or control cohort. To conduct the test, a group file with the qualifying variants was created for each of the eight genes (*CACNA1A*, *CACNA1B*, *CACNA1C*, *CACNA1D*, *CACNA1E*, *CACNA1G*, *CACNA1H* and *CACNA1I*). Of note, *CACNA1S* was excluded from the analysis as it encodes the pore-forming Ca_V_1.1 α_1S_ subunit that is exclusively expressed in skeletal muscle, so not in the brain and *CACNA1F* was excluded as this gene is located on the X-chromosome, and TRAPD is currently not configured to test non-autosomal chromosomes.

We performed gene-based burden testing for all single-point variants in each cohort. The following steps, in brief, were performed: (1) variants for each *CACNA1x* gene in the case group were compiled into a “SNP file”, (2) a Python script was used to interrogate the VCFs and count the occurrence of variants in each gene in both the case and the control cohorts independently. This generated variant count data for each gene, and (3) the one-sided Fisher exact test was used on the allele count tables to identify the probability of excess in the number of allele counts in cases relative to controls (i.e. the statistical significance of the burden). (4) The one-sided Fisher exact test was used on the subject count tables to identify the probability of excess in the number of subjects with variants in cases relative to controls (i.e., the statistical significance of the burden). *P*-values < 6.25 × 10^−3^ were considered significant (Bonferroni corrected for testing 8 genes). Odd ratios were calculated to assess the magnitude of the burden effect. Genes exhibiting statistically significant burden in HM from the Australian discovery cohort were also tested in the Dutch replication cohort.

## Results

### Single-Variant Analysis

No clear pathogenic mutations in *CACNA1x* genes were identified from the WES data in patients from either the Australian or the Dutch cohorts. However, the number of variants in *CACNA1x* genes prompted us to perform burden testing. In the Australian cohort, we identified 79 different missense variants in the eight *CACNA1x* genes examined in the 184 HM patient group from Australia (Supplementary Table 1). All but seven of the variants had been previously identified (i.e., they have an rs number in dbSNP). The seven novel variants were all single-case across multiple different *CACNA1x* genes. In the Dutch cohort, four different variants were identified in *CACNA1I* and ten in *CACNA1H*; all of which had been previously identified (Supplementary Table 2). Although some missense variants in *CACNA1x* genes were predicted to have a pathogenic potential, there was not enough evidence for causality in a monogenic manner such as has been shown for the three well-known HM genes. The results of the individual variant analyses indicate the existence of many variants across *CACNA1x* genes that in combination could plausibly confer increased susceptibility to HM, especially when considered collectively using burden analysis.

### Multiple-Variant Burden Testing of *CACNA1x* Genes

**Fig. 2:**
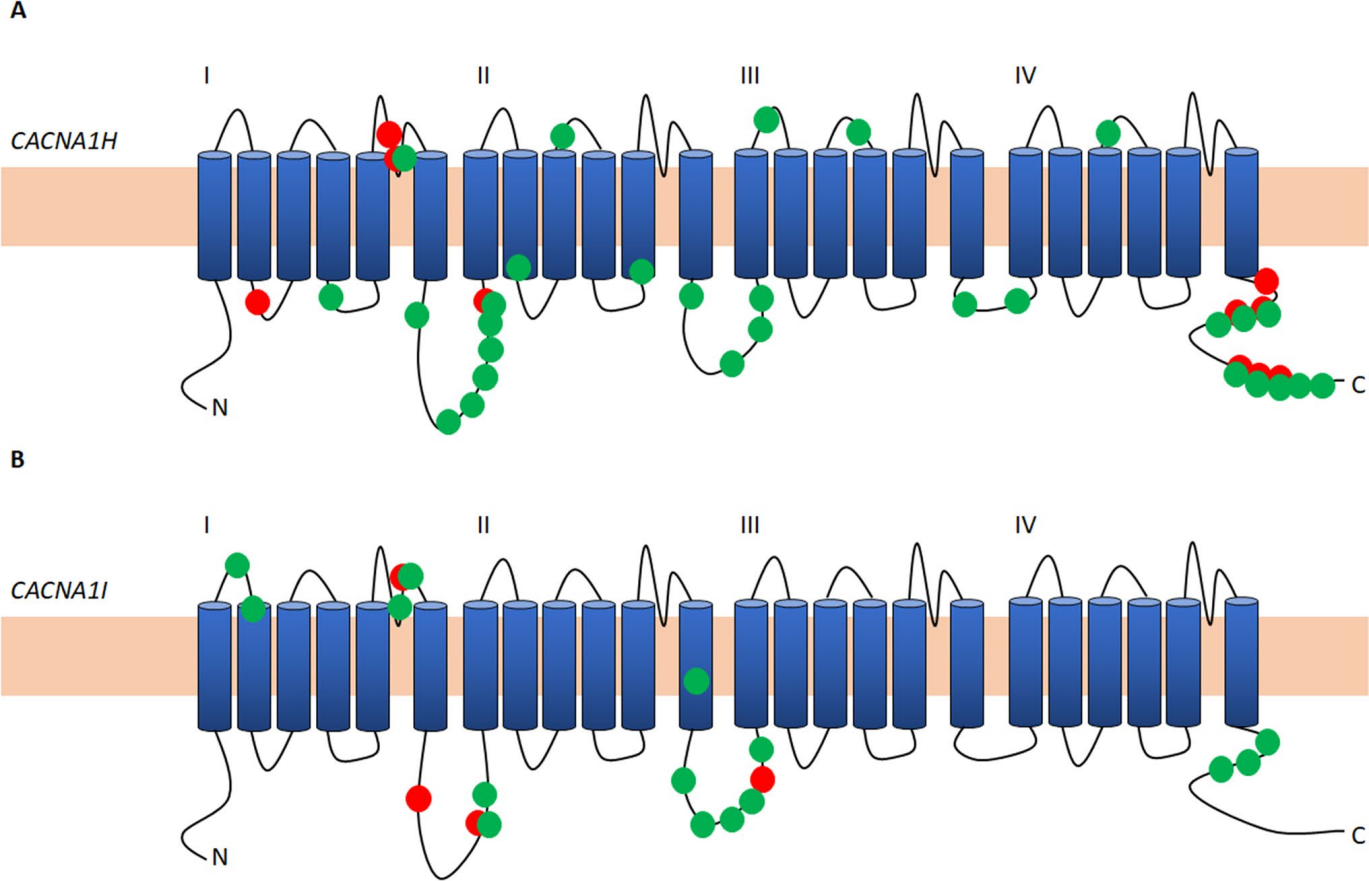
Schematic representation of the α1 subunit, with the position of identified variants in this study, of the Ca_V_3.2 and the Ca_V_3.3 channels, encoded by the *CACNA1H* (**A**) and *CACNA1I* (**B**), respectively. Variants identified in the Australian HM cohort are depicted with a green dot, and variants identified in the Dutch HM cohort are depicted with a red dot

Considering the results from the individual variant analysis, a multiple-variant burden analysis was performed to test whether there is an over-representation of *CACNA1x* missense variants in HM compared to controls. As shown in Table 2, in the Australian HM cohort, this analysis revealed a significantly increased burden of missense variants for those with HM in *CACNA1E* (*p* = 1.3 × 10^−4^), *CACNA1H* (*p* < 2.2 × 10^−16^) and *CACNA1I* (*p* < 2.2 × 10^−16^). For the Dutch cohort, this replicated for *CACNA1H* (*p* = 3.5 × 10^−8^, *p*_cor_ = 1.04 × 10^−7^) and C*ACNA1I* (*p* = 0.019, *p*_cor_ = 0.056), but not *CACNA1E* (*p* = 0.85), albeit that *CACNA1I* did not remain significant after correction for multiple testing. In addition, the number of subjects carrying a variant was also higher cases in *CACNA1E* (*p* = 6.2 × 10^−3^), *CACNA1H* (*p* < 2.2 × 10^−16^) and *CACNA1I* (*p* < 2.2 × 10^−16^) (Table 2). The results showed evidence of replication in the Dutch cohort for *CACNA1H* (*p* = 1.2 × 10^−2^; *p*_cor_ = 3.6 × 10^−2^) and *CACNA1I* (*p* = 4.4 × 10^−2^; *p*_cor_ = 0.13), but not for *CACNA1E* (*p* = 0.88), albeit *CACNA1I* did not remain significant after correction for multiple testing. All but four variants were outside the transmembrane domains that are typically affected in case of a pathogenic mutation in either Ca_V_ channel (Fig. 2).

**Table 2:** Missense variant burden in the discovery cohort consisting of Australian cases and gnomAD controls

Gene	Gene length (bp)	Case count HET	Case count HOM	Control count	Case allele count	Control allele count	*p*-value subjects^a^	*p*-value allele counts^a^
*CACNA1A*	8392	54	1	23,977	60	26,841	1	1
*CACNA1B*	9790	6	0	1815	7	1819	0.54	0.39
*CACNA1C*	8425	4	0	1221	4	1225	0.56	0.57
*CACNA1D*	7636	4	0	2014	4	2024	0.89	0.90
*CACNA1E*	7067	109	41	29,644	228	53,792	6.21 × 10^−3^*	1.30 × 10^−4^*
*CACNA1G*	7648	12	0	1768	12	1770	1.33 × 10^−2^	1.65 × 10^−2^
*CACNA1H*	8084	155	88	20,456	637	22,942	< 2.2 × 10^−16^*	< 2.2 × 10^−16^*
*CACNA1I*	10,004	126	44	4652	277	4694	< 2.2 × 10^−16^*	< 2.2 × 10^−16^*

Case count, number of cases with at least one variant (Het, heterozygous variant; Hom, homozygous variant); Control count, number of controls derived from gnomAD with a variant; Case allele count, total allele count in cases; Control allele count, total allele count in controls derived from gnomAD

^*^Significant results

^a^*p*-values < 6.25 × 10^−3^ were considered significant (Bonferroni corrected for testing 8 genes)

## Discussion

Here we used WES data from 184 suspected HM patients from an Australian clinically referred cohort and compared these to the publicly available gnomAD control dataset using TRAPD, finding that *CACNA1E*, *CACNA1I* and *CACNA1H* missense variants were more prevalent in cases. Furthermore, we show evidence for replication of these findings for *CACNA1H* and *CACNA1I* in a Dutch clinical HM cohort. This finding emphasises that although the cohorts differ in terms of inclusion criteria, the results are transferable to both groups.

In the general population, females are overrepresented in most forms of migraine including hemiplegic migraine. The overall female to male sex ratio in our HM cohort was ~ 1.97:1. This observed difference in prevalence will in part be explained by the fact that females are more inclined to consult a physician and thus are diagnosed earlier and more often than males [40]. We cannot rule out that there is any sexual dimorphic effect at any of the *CACNA1x* genes (i.e., a sex bias in gene function), but we consider this a minor factor compared to the ascertainment bias.

We have hypothesised that HM may not be autosomal dominant in a substantial proportion of cases, but rather is genetically a more complex trait. The difficulty in confirming this hypothesis lies in how to identify such variants, as they are neither identified by gene association approaches, nor in genome-wide association studies (GWAS). In order to identify such variants, we have used a methodology adapted from a TRAPD analysis. The methodology has proven itself by the identification of functional genetic variants in idiopathic hypogonadotropic hypogonadism [39]. By slightly adapting the method, we have been able to investigate all missense variance and thereby determine the variant and subject burden. Similarly, our results show that the accumulation of missense variants in *CACNA1H* and *CACNA1I* plays a role in HM.

*CACNA1H* and *CACNA1I* encode the α1 subunits of Ca_V_3.2 and Ca_V_3.3 LVA T-type calcium channels, respectively (Fig. 1) [22, 23]. *CACNA1H* is expressed ubiquitously, whereas *CACNA1I* is predominantly expressed in the brain [25]. Ca_V_3.2 and Ca_V_3.3 LVA T-type calcium channels [41], which open by only a small membrane depolarization, coupled with their tonic inactivation near resting membrane potential, underlie the spike/rebound bursting phenomenon seen with many types of neurons [42, 43]. These channels are localised at presynaptic nerve terminals [44] where they control synaptic transmission by directly triggering the release of neurotransmitters [45–47]. Inactivation of *Cacna1h* in mice led to decreased nociceptive signalling [48, 49] and several neurological symptoms [50, 51], whereas *Cacna1i* knock-out mice, and also *Cacna1i*/*Cacna1h* double knockout mutants, show implications for sleep rhythmogenesis [52]. T-type channels are important for human physiology, so mutations in these channels may lead, at least in theory, to channelopathies with clinical manifestations resulting from aberrant biophysical characteristics and/or cell surface trafficking issues of channels due to a gain or loss of channel function. Indeed, specific missense variants in *CACNA1H* have been implicated in a range of human conditions [50], including autism spectrum disorders [53] and amyotrophic lateral sclerosis [54]. Many missense variants in the human *CACNA1H* gene have been reported in patients presenting with a range of epilepsy syndromes [50], so the gene was labelled a risk gene for idiopathic generalised epilepsies (38). Functional analyses in embryonic kidney cells, however, revealed that the variants in *CACNA1H* generally produce mild biophysical changes and in some cases do not alter the gating of the channel and variants do not segregate with the phenotype [50]. Hence, their contribution to human epilepsies should be debated, as was recently suggested [28]. In line with this suggestion, it is not unexpected that *CACNA1H* variants identified in HM patients also not solely cause disease, although a burden of variants in this gene can still contribute to HM risk. Similarly, *CACNA1I* loss-of-function variants were identified that alter the gating properties of Ca_V_3.3 channels, disrupt neuronal excitability and network activity, and have been associated with risk of developing schizophrenia and a range of neurodevelopmental disorders featuring developmental delay and epilepsy [55, 56]. Moreover, using patch-clamp electrophysiology, we have shown various functional alterations of channel activity for selected Ca_v_3.3 rare variants, providing further evidence that *CACNA1I* may play a role in the development of HM [57].

Hence, the most likely scenario is that an increased burden of missense variants in *CACNA1H* and *CACNA1I* acts as a genetic modifier of disease risk. Such a modification of risk is not different when reviewing mutations that have been identified in some HM patients in a number of genes, including *PRRT2* [12], *PNKD* [58], *SLC4A4* [59], *SLC1A3* [60] and *SLC2A1* [61], that are primarily associated with movement or solute transport disorders.

Our study has some limitations. First of all, contrary to what is commonly undertaken in genetics, we considered both rare and common variants as an overarching burden of missense variants in this study. To support the validity of this approach, we used the Dutch replication cohort to validate findings from the Australian cohort. Further replication efforts in other independent cohorts would be of benefit in future studies of these genes. Secondly, as this study is the first of its kind, we narrowed the genes targeted to *CACNA1x* ion channels, due to known association of genes of this family with HM. However, the burden of variants in additional genes is likely to play a role in determining HM disease risk. Thirdly, we have used summary statistics for the controls that prevented us to compare ancestry of cases and controls together, although we ensured that both cases and controls were of European ancestry. Finally, the use of the gnomAD population as a control cohort means that we are not comparing truly matched populations. Both in our case cohorts and the gnomAD cohort, there are slightly more female than male participants, that is, the female to male ratio for the cases is ~ 1.97:1, and for the controls, it is ~ 1.27:1, which may result in a slight confounding effect, as does differences in environmental and cultural differences that could not be controlled in our study.

## Conclusion

This study provides evidence that increased burden of missense variants in the amount of variants and the number of subjects carrying a variant in *CACNA1H* and *CACNA1I* exists for HM, and that these genes can modify HM disease risk, supporting more complex types of heritability for HM, in addition to the strictly monogenic forms.

## Supplementary Information

Below is the link to the electronic supplementary material.

Supplementary file1 (XLSX 80 KB)

Supplementary file2 (XLSX 16 KB)

## Data Availability

The data used and/or analysed during the current study are available from the corresponding author on reasonable request.
